# Resource instability undermines predictable plasticity‐mediated morphological responses to diet in a postglacial fish

**DOI:** 10.1002/ece3.10932

**Published:** 2024-02-08

**Authors:** J. Peter Koene, Colin E. Adams

**Affiliations:** ^1^ Scottish Centre for Ecology and the Natural Environment (SCENE), School of Biodiversity, One Health and Veterinary Medicine University of Glasgow Glasgow UK

**Keywords:** brown trout, phenotypic plasticity, plastic rescue, resource instability, stable isotopic niche, trophic morphology

## Abstract

Phenotypic plasticity has been presented as a potential rapid‐response mechanism with which organisms may confront swift environmental change and increasing instability. Among the many difficulties potentially facing freshwater fishes in recently glaciated ecosystems is that of invertebrate prey communities becoming significantly altered in species composition and relative abundance. To test how the rapidity of diet resource change may affect phenotypic responses during development, we subjected juvenile brown trout to pelagic‐type or littoral‐type diets that alternated either daily, sub‐seasonally, or not at all over a single growth season. The proportional intake of each diet was traced with stable isotopes of carbon and nitrogen and modelled with morphometric data on head and jaw shape. While those trout exposed to a single diet type developed predictable morphologies associated with pelagic or littoral foragers, those raised on alternating diets expressed more unpredictable morphologies. With extreme (daily) or even sub‐seasonal (monthly) resource instability, the association of diet type with the phenotype was overwhelmed, calling into question the efficacy of plasticity as a means of adaptation to environments with rapidly fluctuating prey resources.

## Graphical abstract

Phenotypic plasticity has been presented as a potential rapid‐response mechanism with which organisms may confront swift environmental change and increasing instability. We subjected juvenile brown trout to diet regimes, traced by stable isotopes of carbon and nitrogen, alternating at various temporal scales. We found that with extreme or even sub‐seasonal resource instability, the association of diet type with the phenotype was overwhelmed, calling into question the efficacy of plasticity as a means of adaptation to environments with rapidly fluctuating prey resources.

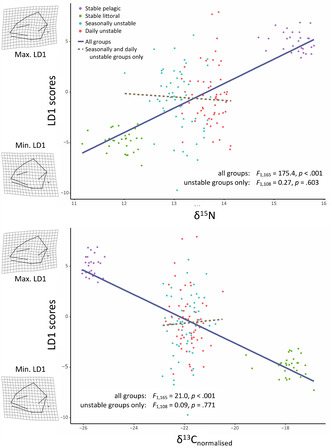

## 1 INTRODUCTION

Considerable intraspecific phenotypic variation is commonly found among fishes of recently glaciated lakes and streams (Carvalho, 1993; Klemetsen, 2013). Fragmented and heterogeneous, such ecosystems present the contrasting ecological opportunities that comprise an important antecedent to emergent phenotypic variation (Dieckmann & Doebeli, 1999; Seehausen, 2007). In the aftermath of the Pleistocene glaciation over the past 12,000–15,000 years, a great variety of available niches became available in recently glaciated systems to the relatively few species able to (re)colonise them (Pabijan et al., 2015). Within species, evolutionary responses to different local environmental conditions may lead to structuring of phenotypic variation between discrete habitats (Bolnick et al., 2011; Koene et al., 2020; Recknagel et al., 2017). For carnivorous fishes, such systems may present ecological contrasts in the form of alternate foraging resources, which may promote characteristic, adaptive phenotypes associated with foraging specialisms (Bush & Adams, 2007; Skúlason et al., 1999). Well‐documented in a range of postglacial fishes, intraspecific alternative behavioural, morphological, physiological, and life‐history phenotypes have been linked to exploitation of different resources (e.g. Bolnick & Ballare, 2020; Hooker et al., 2017; Ide et al., 2011; Markevich et al., 2018; Skúlason et al., 1999; Thomas et al., 2019). Commonly reported in many postglacial fish species is intraspecific divergence into specialist pelagic and benthic forms feeding on zooplankton and macroinvertebrates, respectively. These are often in sympatry under disruptive selection (McPhee et al., 2012; Smith & Skúlason, 1996; Vonlanthen et al., 2009), and with each group displaying its own phenotypic peculiarities (e.g. Fraser et al., 2008; Garduño‐Paz & Adams, 2010; Hendry et al., 2009; Præbel et al., 2013).

Phenotypic plasticity, the developmental process through which a given genotype may express alternative phenotypes (West‐Eberhard, 1989), is considered a vehicle that can facilitate rapid divergence of adaptive phenotypes across differing ecological niches presenting alternative foraging resources (*vide* Levis & Pfennig, 2016). By expediting the tailoring of the phenotype to the local environment, plasticity may be critical to a species' success at colonising new habitats or adapting to environmental change (Januszkiewicz & Robinson, 2007; Parsons & Robinson, 2006). The importance of plastic ‘rescue’ has become particularly relevant in confronting rapid anthropogenic environmental change (Chevin et al., 2013; Hollander et al., 2015; Sih, 2013; Snell‐Rood et al., 2018), particularly as a buffer that allows alternative fitness peaks within changing adaptive landscapes to be bridged, while buying time for adaptive evolution (Harmon & Pfennig, 2021). Research in recent decades has increasingly noted the role of plasticity in facilitating phenotypic responses specifically to climate change (*vide* Merilä & Hendry, 2014; Fox et al., 2019). Beyond the direct effects of increased temperatures on the physiology, growth, mortality, etc. of fishes in recently glaciated lakes (*vide* Solomon, 2008; Vornanen et al., 2014; Santiago et al., 2016), climate change is predicted rapidly impact local environments: changes to the thermal dynamics of lakes can have profound consequences for nutrient upwelling and, in turn, primary production and food web dynamics (Sahoo et al., 2016; Woodward et al., 2010); invertebrate prey communities may become significantly altered in species composition and relative abundance of component populations (Cao et al., 2020; Hart & Gotelli, 2011). Behaviour and foraging activity of fishes have been shown in natural lake experiments to change significantly under different temperature conditions (Biro et al., 2007; Sánchez‐Hernández et al., 2021). Morphological responses to changing foraging resources, potentially effected through phenotypic plasticity, have already been noted in a tropical fish species (Cardozo et al., 2021); and phenotypic responses to a single rapid change in rearing habitat, with an implied impact on available food resources, have been investigated experimentally in a fish species occupying previously glaciated systems (Sánchez‐González & Nicieza, 2017). The rapid adoption of characteristic head and jaw morphology associated with foraging, and mediated by plasticity, has been well‐established experimentally in many postglacial fishes through diet‐induced polymorphisms (e.g., Adams & Huntingford, 2004; Andersson, 2003; Cucherousset et al., 2011; Day & McPhail, 1996; Garduño‐Paz et al., 2020; Parsons & Robinson, 2007); however, these experiments presented temporally stable access to foraging resources over a growth season, and none introduced an element of resource fluctuation.The temporal scale of stability of resource access that is required for a predictable plastic response during ontogeny is not currently understood.

To investigate the potential effects of environmental instability on plasticity‐mediated head and jaw morphology, we designed an experiment using young‐of‐the‐year brown trout (*Salmo trutta* L.), in which pelagic‐type and littoral‐type diets, each requiring distinct foraging methods, alternated at different intervals to simulate consistent (acting as controls), sub‐seasonal (monthly), or severely unstable (daily) access to resource types. A common species inhabiting recently glaciated lakes and native to most of northern Eurasia, brown trout is known to exhibit both high degrees of morphological plasticity (Vehanen & Huusko, 2011) and sensitive morphological responses to local environments (Koene et al., 2020). Yet the species faces threats due to rapid climate change, with concern about adaptability (Jensen et al., 2008). Thus, we tested the hypotheses (a) that the expressed morphology of fish would be predictable and contrasting in the two constant‐exposure diet control groups and (b) that the groups exposed to an alternating foraging environment would express group‐mean head shapes intermediate between specialist littoral and pelagic feeders. However, we further predict (c) that the rapidity of resource change will prevent individuals in the unstable diet groups from expressing predictable phenotypes.

## 2 METHODS

### 2.1 Sample collection and husbandry

Juvenile brown trout (aged 0+, young‐of‐the‐year, *n* = 250) were collected in November 2017 using a DC 500 W battery‐powered backpack electro‐fishing kit (E‐fish (UK) Ltd.) and a single‐pass approach from four streams in the Loch Lomond catchment, Scotland, UK: Boquhan Burn (56°3′33″ N, 4°20′13″ W), Blane Water (55°59′20″ N, 4°19′32″ W), Ross Burn (56°8′12″ N, 4°37′22″ W), and Wood Burn (56°8′18″ N, 4°37′46″ W). The fish were taken to the Scottish Centre for Ecology and the Natural Environment (SCENE) at Loch Lomond, where they were anaesthetised with a 33.5 mg L^−1^ benzocaine solution (from 4% stock prepared in ethanol) (Zahl et al., 2012), measured for mass and fork length, and marked with uniquely devised patterns of visible implant elastomer tags (Northwest Marine Technology Inc.) so that specimens could later be identified individually. Trout from each site of origin were randomly allocated one of eight identical, cylindrical, individual flow‐through (ca. 10 L min^−1^ turnover) tanks measuring 76 cm internal diameter by 50 cm high, filled to 120 L, so that each tank had roughly equal numbers of trout from each stream and 31 or 32 fish in total. Water was supplied to the tank from Loch Lomond at ambient temperatures (min. spring: 4°C; max. summer: 20°C) and with a pronounced directional current. Throughout the experiment, the natural photoperiod for this latitude (56° N) was imitated with artificial lighting. Each tank was provided with an air stone and no other ornamentation. During an acclimation period and over winter (105 days), all fish were fed a maintenance diet of frozen bloodworm once per day; thereafter, from March 2018, fish were fed an experimental diet once per day for 245 days (8 months to the end of October). Fish that died or appeared ill were excluded from analyses.

### 2.2 Experimental protocol

Tanks were assigned one of four treatment conditions based on diet: (1) pelagic‐type prey diet exclusively (‘stable pelagic’, control), (2) littoral‐type prey diet exclusively (‘stable littoral’, control), (3) pelagic‐type and littoral‐type diets alternating monthly (‘seasonally unstable’), or (4) pelagic‐type and littoral‐type diets alternating daily (‘daily unstable’). There were two replicates of each treatment group. The pelagic‐type diet consisted of frozen *Mysis* (Tropical Marine Centre, Ltd.: min. 10.2% crude protein, min 1.1% crude fat, max. 0.4% crude fibre, 𝛿^15^N = 11.91, and 𝛿^13^C = −27.18), thawed, mixed with lake water, and poured slowly into the top of the water column to be carried by the current. The littoral‐type diet was frozen bloodworm (Tropical Marine Centre, Ltd.: min 4.5% crude protein, min. 0.2% crude fat, max. 0.7% crude fibre, 𝛿^15^N = 10.53, 𝛿^13^C = −15.97), thawed, embedded within ca. 20 cm × 15 cm patches of artificial grass (B&Q plc ‘Banbury’) to prevent bloodworms from floating around the tanks, refrozen, and placed on the tank bottom, two grass patches per tank. All fish were fed to excess, evidenced by leftover food, once per day, mid‐afternoon.

**FIGURE 1:**
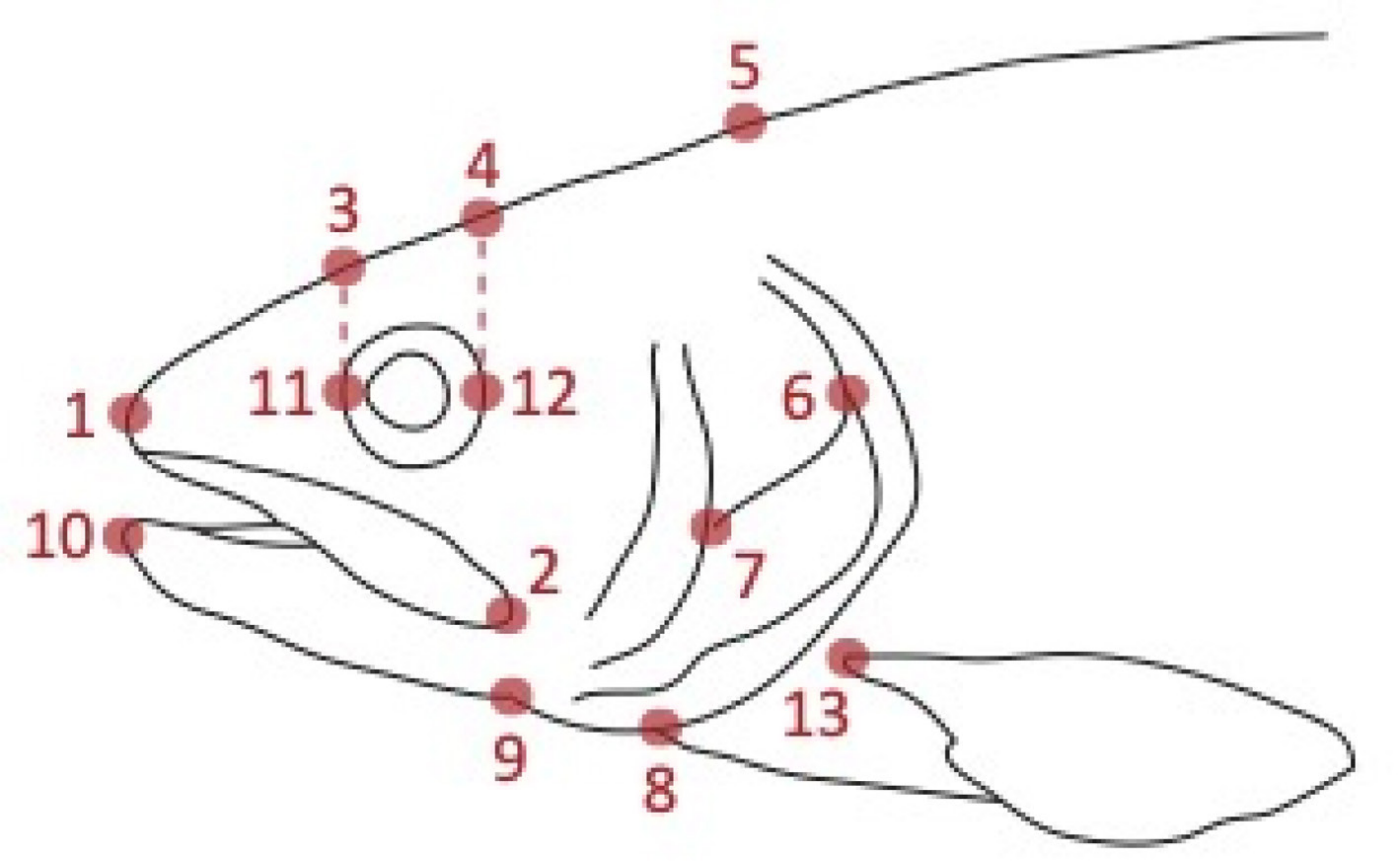
Landmark positions on heads of brown trout for geometric morphometric analysis: (1) tip of the snout; (2) posterior tip of maxilla; (3–4) superior of the cranium respectively perpendicular to 11 and 12, alignments indicated with broken lines; (5) superior posterior of the cranium; (6) anterior intersection of opercula and subopercule; (7) inferior intersection of the opercula and preopercule; (8) ventral margin of the opercula; (9) ventral margin of the mandible; (10) anterior tip of the mandible; (11–12) anterior and posterior of eye); (13) superior insertion of the pectoral fin.

Included in the analyses were 224 fish: stable pelagic, *n* = 51; stable littoral, *n* = 61; seasonally unstable, *n* = 55; and daily unstable, *n* = 57. After 245 days of treatment, all fish were euthanised and measured for mass and fork length. Digital photographs were taken for each specimen, immediately after death, of the left side of the head. Images were made into thin plate spline (TPS) files with tpsUtil (Rohlf, 2017b); scale calibration and landmarking of 13 homologous points describing head shape were executed by the same researcher using tpsDig2 (Rohlf, 2017a; Figure 1).

Samples of food sources and small sections of white muscle tissue from the upper flank were taken from each specimen, dried, pulverised, and sent to the Natural Environment Research Council Life Sciences Mass Spectrometry Facility (NERC LSMASF) in East Kilbride, Scotland, UK, for analysis of stable isotopes of nitrogen and carbon. Samples were analysed using a Vario‐Pyro Cube elemental analyser (Elementar) coupled with a Delta Plus XP isotope ratio mass spectrometer (Thermo Electron) and laboratory standards (gelatine and amino acid–gelatine mixtures) MSAG2, M2 and SAAG2, and international standard (glutamic acid) USGS40.

### 2.3 Statistical analyses

All statistical analyses were carried out using *R* v.3.6.1 (R Core Team, 2019). Normality of variance was examined with Q‐Q plots for fork length, mass, stable isotope data, and C:N ratios. Specific growth rates (SGR) were calculated using the formula of Hopkins (1992):

$$\mathrm{SGR}=\left(\mathrm{Ln}\left(m_{t}\right)–\mathrm{Ln}\left(m_{i}\right)\right)/t,$$

in which Ln(*m*_t_) and Ln(*m*_*i*_) are the natural logarithms of the mass in grams of fish at the end and beginning of the experiment, respectively; and *t* is the duration of the experiment in days.

The effects of treatment on both SGRs were tested with ANOVA. The body condition of all specimens at the beginning and end of the experiment was inferred as residual mass from linear models of log‐transformed body mass on log‐transformed fork length. The effects of treatment and initial residual mass on body condition at the end of the experiment were tested with ANOVA.

C:N ratios are a good indicator of lipid content in consumers, which can affect 𝛿^13^C values (Post et al., 2007; *vide* Table 1). Therefore, the effect of treatment on C:N ratios was tested with ANOVA, and differences in C:N ratios between groups were tested with Waller–Duncan's post hoc with Bayesian approximation. Any such differences between treatment groups were then accounted for mathematically in subsequent analyses by determining normalised 𝛿^13^C for all treatment groups, following the formula for aquatic animals (Post et al., 2007):

$${\text{34𝛿}}^{13}C_{\text{normalised}}={\text{34𝛿}}^{13}C_{\text{untreated}}–3.32+0.99\times C:N.$$

The δ^13^C_normalised_ values were used in preference to δ^13^C_untreated_ in all subsequent analyses.

**TABLE 1:** Four treatment group means (‰) ± SD of δ^15^N and δ^13^C, before and after normalisation, and the ratio of δ^13^C_untreated_ to δ^15^N.

	δ^15^N	δ^13^C_untreated_	C:N	δ^13^C_normalised_
Stbl. pel.	15.42 ± 0.25	−25.70 ± 0.23	3.58 ± 0.12	−25.47 ± 0.18
Stbl. litt.	11.93 ± 0.27	−17.90 ± 0.48	3.43 ± 0.08	−17.82 ± 0.48
Seas. unstbl.	13.09 ± 0.34	−22.00 ± 0.49	3.47 ± 0.12	−21.89 ± 0.50
Daily unstbl.	13.70 ± 0.27	−21.84 ± 0.45	3.39 ± 0.07	−21.80 ± 0.44

Isotope fractionations were estimated by subtracting the diet source 𝛿^13^C and 𝛿^15^N values from the 𝛿^13^C and 𝛿^15^N values of each individual trout specimen (Newton, 2016). For the seasonally and daily unstable groups, an assumption of a 50:50 pelagic: littoral diet was made for the purposes of the estimation of fractionation. ANOVA was used to test treatment‐group effects on fractionation, and differences between groups were tested with the Waller–Duncan's post hoc test.

To determine possible differences in the group isotopic niche, defined as the area in multivariate space in which axes represent relative proportions of isotopically distinct elements incorporated into the muscle tissues for each treatment group (Newsome et al., 2007), the effect of treatment on 𝛿^15^N and 𝛿^13^C together was tested with MANOVA. The treatment effect on individual isotopes was tested with ANOVAs, with Waller–Duncan's post hoc testing for differences between pairs of treatment groups. Isotopic niche size, given as area, ‰^2^, from measurements of 𝛿^15^N ‰ and 𝛿^13^C ‰, were quantified using standard ellipse areas (SEA_b_), calculated in the *R*‐package ‘SIBER v.2.1.3’ (Jackson et al., 2011) for each treatment group, using the Bayesian methodology and accounting for 95% of posterior variability. This package was also used to calculate average Euclidean distances between SEA_b_ centroids (i.e., the mean 𝛿^15^N and 𝛿^13^C values for each group) as measures of differentiation between niches and to calculate the overlap of SEA_b_ (i.e., the percentage of the stable isotopic niche of the daily unstable group that overlaps that of the seasonally unstable group), which together represent packing within trophic niche space (Layman et al., 2007).

Differences in orientation, position, and isometric size of head images were accounted for with a generalised Procrustes analysis (Mitteroecker & Gunz, 2009) in the *R*‐package ‘Geomorph v.3.1.2’ (Adams et al., 2019). The effects of Procrustes centroid size and treatment group, plus their possible interaction, on head shape were tested using Procrustes ANOVA with 1000‐round randomised residual permutation procedures in ‘Geomorph’ and ‘RRPP v.0.4.2’ (Collyer & Adams, 2019). Procrustes centroid size and treatment group both exerted a significant influence on trout head shape (centroid size: *F*_1,226_ = 26.4, *R* ^2^ = .1, *p* < .001; treatment group: *F*_3,226_ = 3.7, *R* ^2^ = .04, *p* < .001), but their interaction did not (*F*_3,226_ = 0.99, *R* ^2^ = .01, *p* = .507), indicating homogeneity of allometric slopes. Shape differences due to allometric effects were, therefore, accounted for by regressing Procrustes coordinates on Procrustes centroid size and using the resulting residuals as size‐independent measures of head shape in subsequent analyses. After confirming normality of variance for the residuals from each Procrustes coordinate with Q‐Q plots, the effectiveness of the account for allometry was confirmed by MANOVA finding no significant effect of fork length on the residuals (*Pillai* = 0.134, *F*_1,222_ = 1.28, *p* = .18). Comparisons of distances between least‐squares (LS) group means, derived from the permutation procedure above and with a 95% confidence level, between treatment groups were made using the ‘pairwise’ function in ‘RRPP’ (Collyer & Adams, 2019).

Linear discriminant analyses (LDAs) of head shape were performed in the *R*‐package ‘MASS’ (Venables & Ripley, 2002) and used to establish an index of morphology between the extremes represented by stable‐diet groups, with which an evaluation could be made of the similarity of individuals from unstable‐diet groups to the extremes. An initial LDA was performed on size‐independent head shape measures from all fish in the stable pelagic and littoral diet control groups. Those individuals that were correctly assigned to their a priori groupings with at least 95% confidence were carried forward to form a clear training set for a second LDA in which the test diet groups, seasonally and daily unstable, were tested against the a priori stable pelagic and littoral diet groups. The difference in mean LD scores between the two unstable diet groups was tested with Welch's two‐sample *t*‐test.

To investigate a relationship between diet and head morphology, linear models of the effects of 𝛿^15^N and 𝛿^13^C on head‐shape LD1 scores were tested with ANOVAs: firstly, on all specimens from all treatment groups, i.e., the reduced LDA set of stable pelagic and littoral diet treatment groups plus the seasonally and daily unstable diet groups, and secondly, on the two unstable diet groups alone.

## 3 RESULTS

**FIGURE 2:**
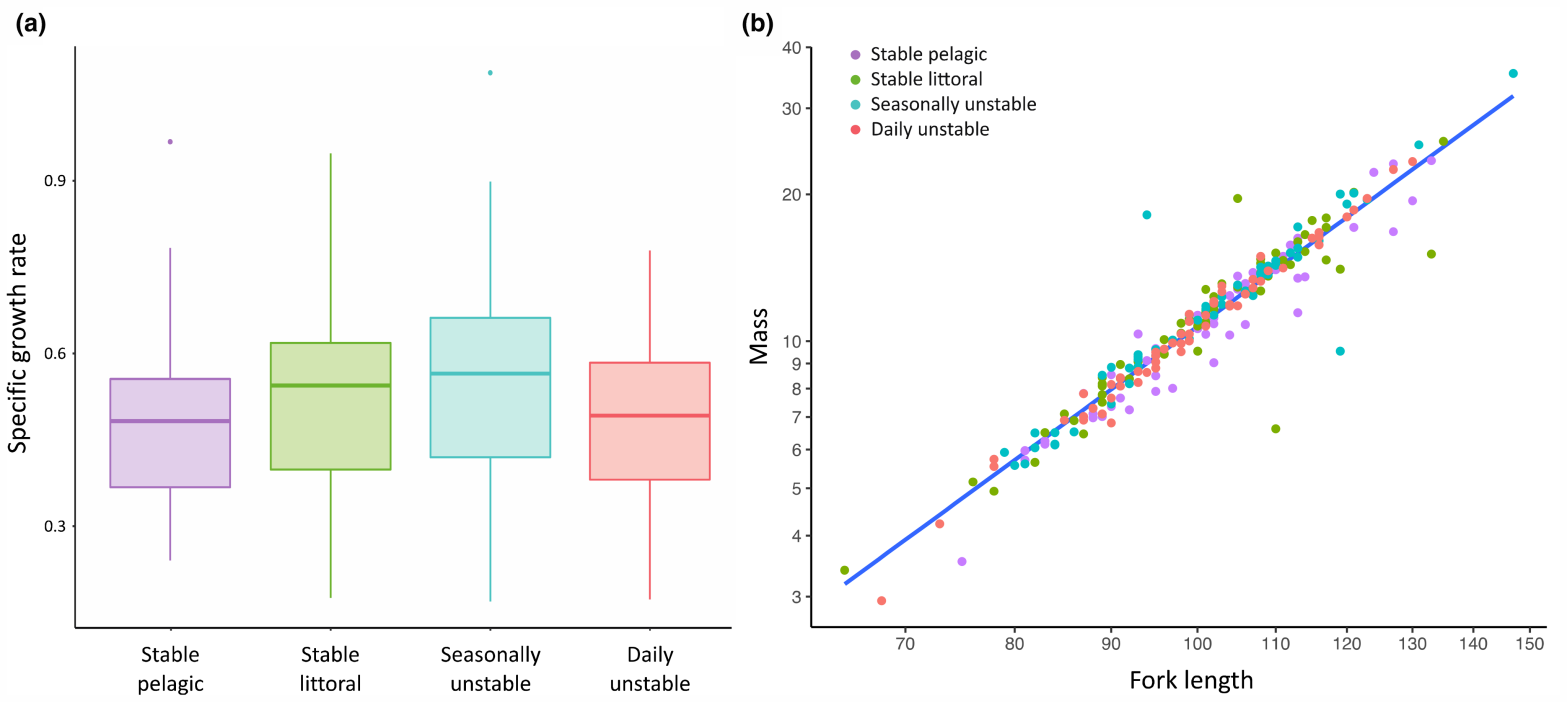
Measures for four treatment groups of brown trout of (a). specific growth rate over 8 months and (b). condition (residual mass) after 245 days of treatment, using log‐transformations of mass (g) and fork length (mm).

**FIGURE 3:**
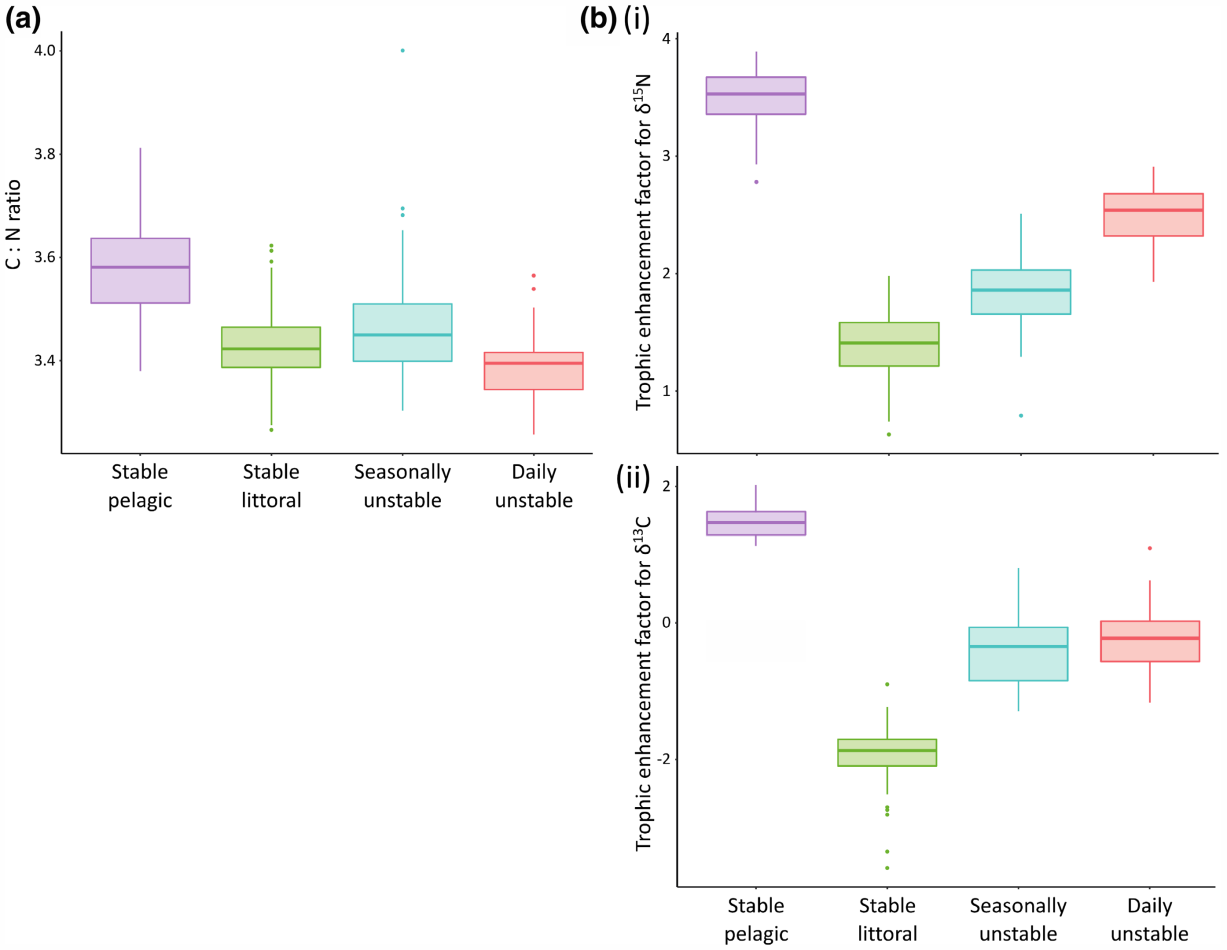
Measures for four treatment groups of brown trout of (a). ratios of carbon to nitrogen stable isotopes and (b). fractionation of (i) δ^15^N and (ii) δ^13^C.

There was no significant effect of treatment on either specific growth rate (*F*_3,220_ = 1.86, $R_{\mathrm{adj}}^{2}$ = 0.01, *p* = .137) or condition (*F*_4,219_ = 2.14, $R_{\mathrm{adj}}^{2}$ = 0.02, *p* = .077) (Figure 2). However, there were treatment effects on C:N ratios (*F*_3,220_ = 39.39, $R_{\mathrm{adj}}^{2}$ = 0.34, *p* < .001), with each group distinct from one another (Figure 3a). Treatment also had a strong effect on the fractionation of nitrogen (*F*_3,220_ = 559.3, $R_{\mathrm{adj}}^{2}$ = 0.883, *p* < .001) and carbon (*F*_3,220_ = 648.4, $R_{\mathrm{adj}}^{2}$ = 0.897, *p* < .001); significant differences were noted between all pairs of groups, except for carbon between the seasonally and daily unstable groups (Figure 3b).

**FIGURE 4:**
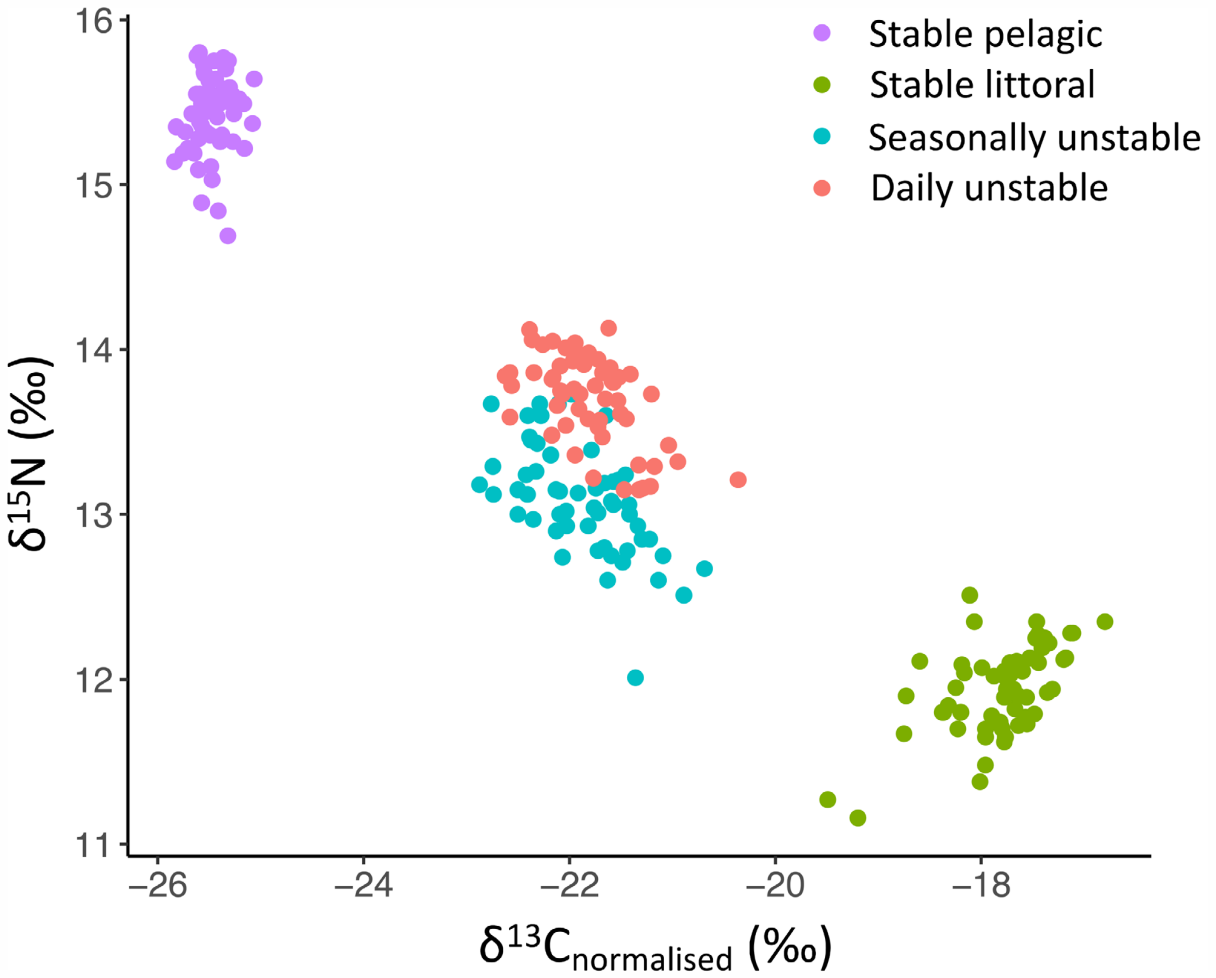
Isospace plot showing δ^15^N and δ^13^C values for individual brown trout, coloured by the treatment group.

**FIGURE 5:**
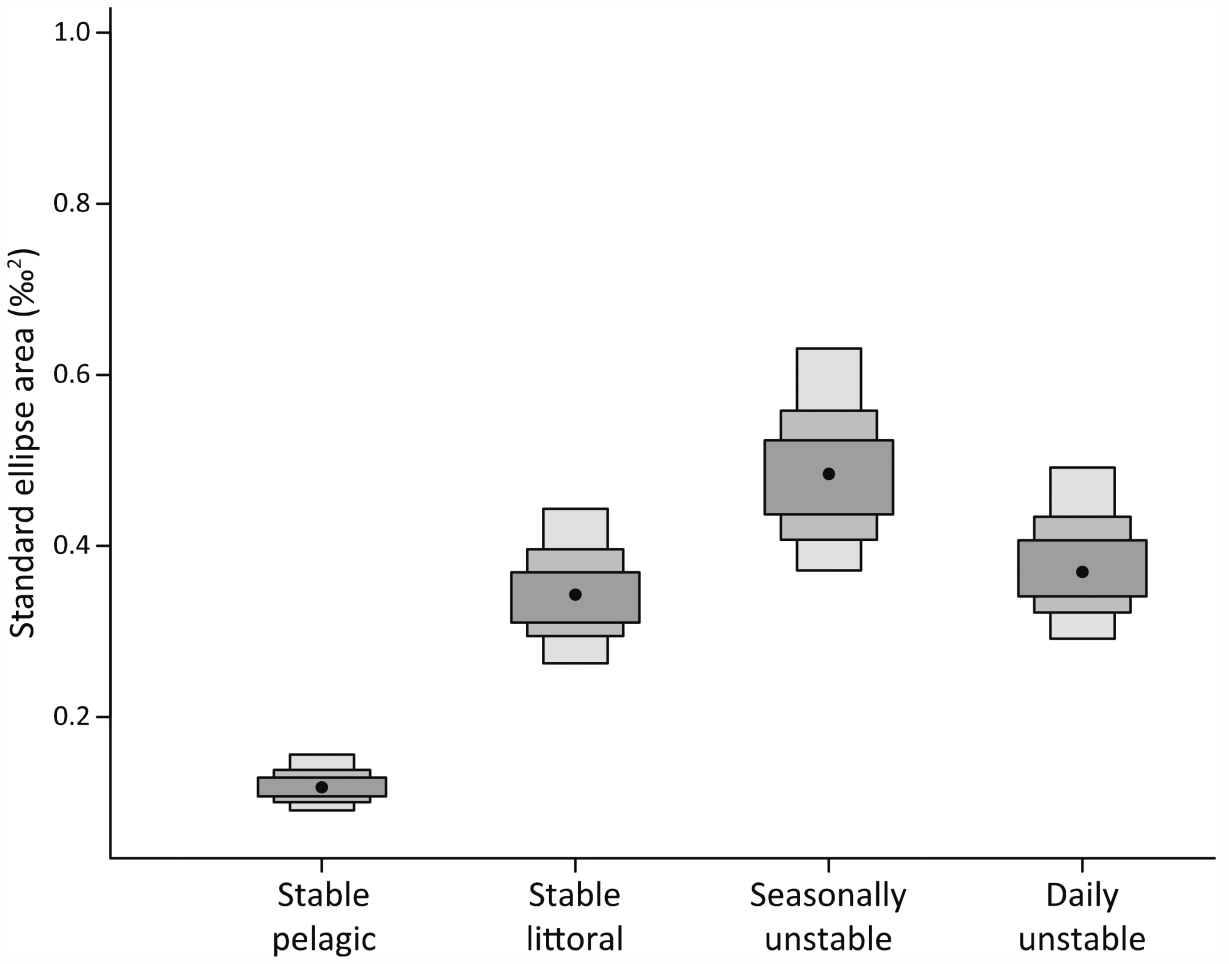
Bayesian standard ellipse areas (SEA_b_) quantifying isotopic niche size by the treatment group. Light grey = 95% credible interval (CI), medium grey = 75% CI, dark grey = 50% CI; black spot = mode.

Measurements of δ^15^N and δ^13^C were significantly affected by treatment (*Pillai* = 1.45, *F*_3,220_ = 193.1, *p* < .001) (Figure 4; Table 2), and niche size (area) also differed between groups (Figure 5). Centroid distances were greatest between the two control groups and smallest between the two unstable diet groups (Table 3). Of the daily unstable diet group's SEA_b_, 41.63 ± 0.74% (mean ± 95% CI) overlapped with the SEA_b_ of the seasonally unstable diet group, indicating a considerable degree of similarity in resource use between these two treatment groups.

**TABLE 2:** Results from ANOVA testing of the effects of four treatments on δ^13^C_normalised_ and δ^15^N, with Waller–Duncan post hoc test results showing group assignations of significant uniqueness.

	*F*_3,220_	$R_{\mathrm{adj}}^{2}$	*p*	Waller‐Duncan test		
				Treatment	Mean (‰)	Assignation
δ^13^C_normalised_	3020	.98	<.001	Stbl. pel.	−25.47 ± 0.18	a
				Stbl. litt.	−17.82 ± 0.48	b
				Seas. unstbl.	−21.89 ± 0.50	c
				Daily unstbl.	−21.80 ± 0.44	c
δ^15^N	1440	.95	<.001	Stbl. pel.	15.42 ± 0.25	a
				Stbl. litt.	11.93 ± 0.27	b
				Seas. unstbl.	13.09 ± 0.34	c
				Daily unstbl.	13.70 ± 0.27	d

**TABLE 3:** Pairwise Euclidean distances between SEA_b_ centroids, as determined by stable isotopes of nitrogen and carbon, of four treatment groups.

	Stbl. pel.	Stbl. litt.	Seas. unstbl.
Stbl. litt.	8.54		
Seas. unstbl.	4.37	4.26	
Daily unstbl.	4.22	4.32	0.63

**FIGURE 6:**
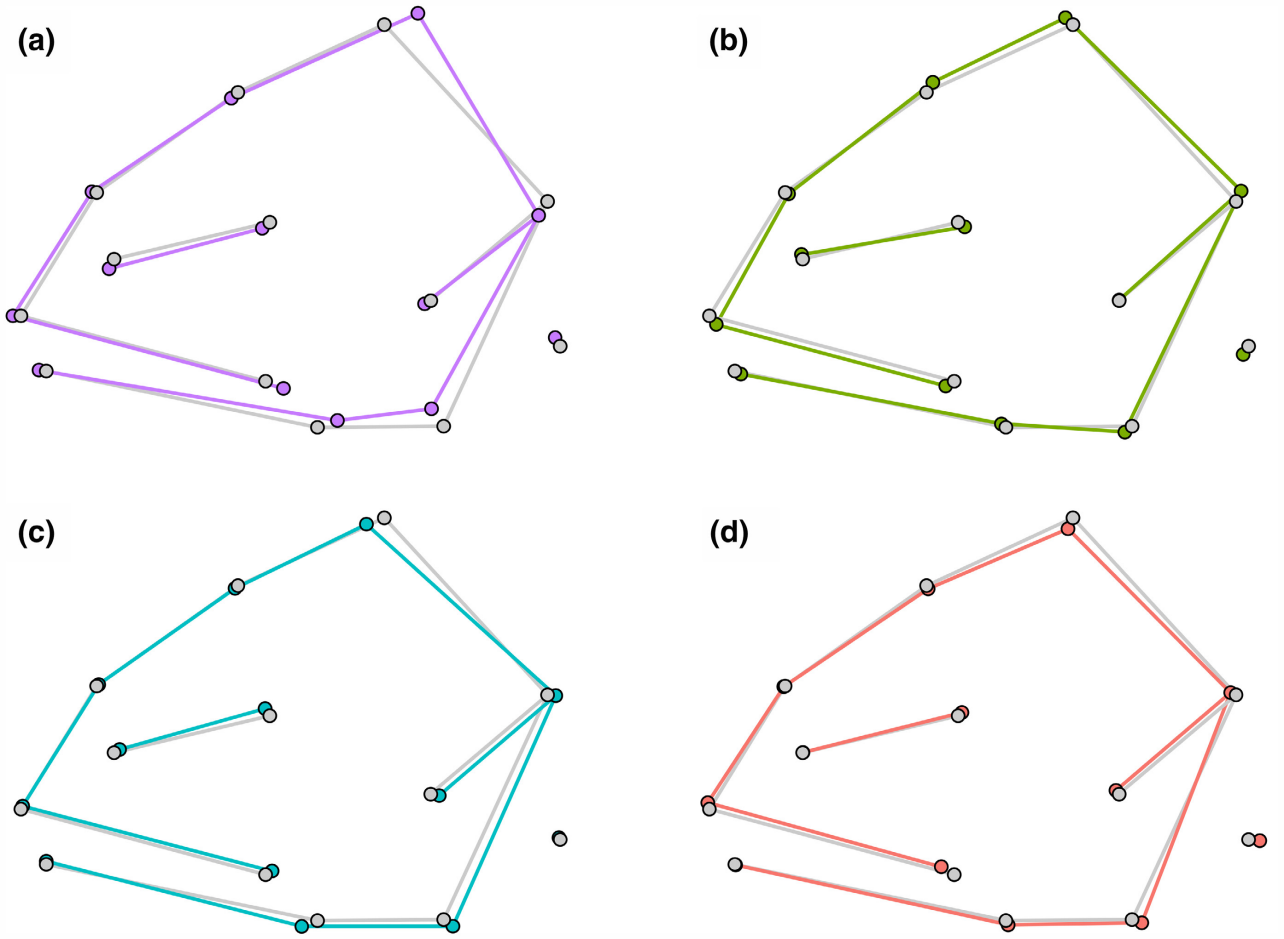
Differences in mean head shape by each treatment group (coloured) compared to overall mean (grey): (a) stable pelagic, (b) stable littoral, (c) seasonally unstable, and (d) daily unstable.

Considering head morphology, pairwise LS means distances were highly significant between all pairs of treatment groups involving the stable pelagic diet group (all *p* = .001). Distance was also significant between the stable littoral and daily unstable diet groups (*p* = .037), but only nearly so between the stable littoral and seasonally unstable diet groups (*p* = .052), and not at all between the two unstable diet groups (*p* = .203) (Table 4). The stable pelagic diet group was characterised by longer heads and jaws, while the stable littoral diet group showed deeper heads in relation to length; the seasonally and daily unstable diet groups were typically intermediate between the control groups (Figure 6).

**TABLE 4:** Pairwise comparisons of LS‐means distances (derived from head shape coordinates) between brown trout from four treatment groups.

Pair	Distance	UCL (95%)	*Z*	*p*
SP:SL	0.021	0.012	6.149	.001
SP:SU	0.023	0.013	6.584	.001
SP:DU	0.021	0.013	5.874	.001
SL:SU	0.012	0.012	1.795	.052
SL:DU	0.012	0.012	1.978	.037
SU:DU	0.010	0.012	0.886	.203

Abbreviations: DU, daily unstable; SL, stable littoral; SP, stable pelagic; SU, seasonally unstable.

The initial LDA of the stable pelagic and littoral diet control groups correctly assigned individuals to their groups with an accuracy of 83.9%. Higher LD1 scores were associated with ‘pelagic’ morphology. Using the 28 stable pelagic and 29 stable littoral individuals correctly assigned with a confidence of ≥95% as a training set, the second LDA revealed the head morphologies of both the seasonally and daily unstable diet groups to be intermediate (LD1‐score means ± SD: −0.78 ± 3.1 and −0.46 ± 2.88, respectively) between that of the stable pelagic diet group (LD1 mean: 5.02 ± 0.99) and of the stable littoral diet group (LD1 mean: −4.84 ± 1.01). The difference between the unstable diet groups was not significant (*t* = 0.562, df = 108.7, *p* = .575).

**FIGURE 7:**
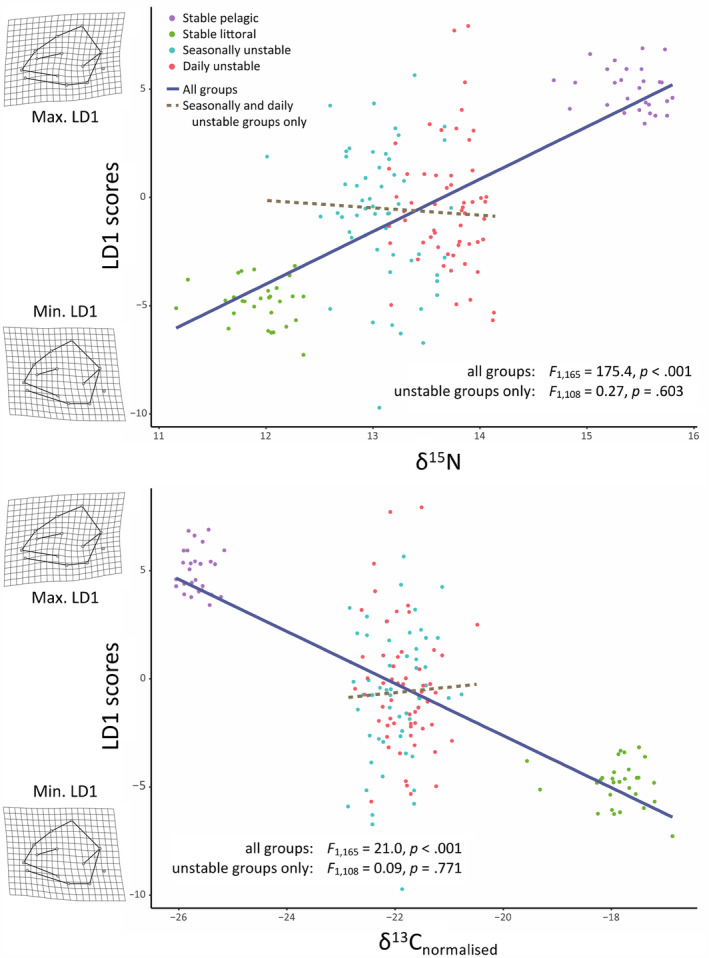
Results from linear models of the effects of a. δ^15^N and b. δ^13^C on individual LD1 scores describing head shape, for all treatment groups together, and for just seasonally and daily unstable groups together.

Overall, there was significant correspondence between head morphology and stable isotope signature (*F*_3,165_ = 66.9, $R_{\mathrm{adj}}^{2}$ = 0.55, *p* < .001), with an increase in LD1 scores (i.e., towards more ‘pelagic’ head shapes) associated with increasing 𝛿^15^N (*F*_1,165_ = 175.4, *p* < .001) and decreasing 𝛿^13^C (*F*_1,165_ = 21.0, *p* < .001) (Figure 7). However, this correspondence disappeared when only the two unstable treatment groups were considered (*F*_3,108_ = 0.52, $R_{\mathrm{adj}}^{2}$ = 0.014, *p* = .666).

## 4 DISCUSSION

Experimental diets were specifically selected because they varied in 𝛿^15^N and 𝛿^13^C, and so it is not surprising that the control groups, each of which consumed exclusively one diet or the other, had the most restricted isotopic niches. The somewhat larger niche of littoral prey diet may be the result of greater isotopic variability among individual prey items or, perhaps, differences in individual trout's rates of assimilation. Generally, however, in this experiment, isotopic niche size is driven by consumer–resource interactions, and, therefore, larger niches, determined by N and C stable isotope space, broadly indicate a more varied diet (Yeakel et al., 2016), necessitated by changes to food availability (Lehmann et al., 2015). In principle, both the seasonally and the daily unstable diet groups had access to both diets in equal proportions over the period of the experiment, yet the seasonally unstable diet group had the larger isotopic niche. As suggested by the fact that the daily unstable diet group's niche was closer (in Euclidean distance) to that of the stable pelagic diet group, despite considerable niche overlap with the seasonally unstable diet group, a preference for the pelagic diet in this group was observed. To some extent, daily unstable diet individuals could specialise in one diet, by consuming more food on 1 day than the next; for the seasonally unstable diet group, this would be much more difficult. However, there was no evidence among any individuals of the daily unstable diet group of full diet specialisation by feeding and fasting on a 2‐day rotation; specialisation was only partial.

Head shapes of the control groups largely conformed to expectations for salmonids: stable pelagic feeders had longer, more gracile heads and jaws, while stable littoral (benthic) feeders had deeper heads in relation to length (e.g., Adams et al., 1998; Koene et al., 2020; Nakano et al., 2020; Præbel et al., 2013). It is clear that under single‐diet treatment conditions, the diet/morphology relationship followed distinct and predictable lines. A certain degree of caution should be applied, however, when interpreting these results as the effect of foraging method on head shape. Although its use as a predictor of lipid content may be questionable, there is an established general association of the C:N ratio with lipid content (e.g., Sweeting et al., 2006; Barnes et al., 2007; Dempsen et al., 2010; but cf. Fagan et al., 2011), and the lower ratio amongst the littoral group suggests relatively lower lipid levels, although this did not affect the relative condition (i.e., residual mass) of the fish. While carbon fractionation followed a classic pattern amongst the stable pelagic feeders (*vide* DeNiro & Epstein, 1978; Vander Zanden & Rasmussen, 2001; Post, 2002), this was not the case for the stable littoral diet group, perhaps suggesting that the two food sources did not represent an equal nutritional value. This is further evidenced by isotopic depletion of nitrogen in the littoral diet group, which is consistent with fish synthesising lipids to compensate for dietary lack (Sakata et al., 1997). However, low δ^15^N in the littoral diet group was not matched by expected lower somatic growth (Buchheister & Latour, 2010; Hesslein et al., 1993), possibly because the fish were fed to excess. Nonetheless, whether through foraging‐method induction or nutrition, it remains clear that the diets played a central role in the plasticity‐mediated differences in head morphology between the two control groups.

The seasonally and daily unstable diet groups had respective consensus head shapes that were intermediate between those of the two control groups, but neither was significantly different from the other, despite those groups' somewhat different isotopic niches. However, the spread of variation within each of the two unstable diet groups was remarkable: even though head‐shape means were intermediate, some individuals displayed morphologies that were more extreme than either of the pelagic or littoral diet groups, while others were intermediate. Furthermore, although the control groups each demonstrated a clear relationship between diet (δ^15^N and δ^13^C) and head morphology, no such relationship was established within the unstable diet groups. Among the unstable diet groups, neither δ^15^N nor δ^13^C was proved able to predict head shape along the pelagic–littoral continuum. Although plastic change appears to have been stimulated by diet among both unstable diet groups, as among the control groups, the connection between diet and the *direction* of plasticity‐mediated morphology collapsed. The uncoupling of diet from head shape occurred regardless of the rapidity of diet change: both an extremely rapid and sub‐seasonal change proved rapid enough to overwhelm the effectiveness of an adaptive plastic response. While this may not be surprising over a short (daily) temporal scale, as may be experienced during severe weather events caused by climate change, that this is also the case over the medium (monthly) scales is an important finding, and it suggests that any sub‐seasonal fluctuation in environmental cues and temporal variability in prey communities may be sufficient to disrupt the effects of the environment on the expressed phenotype.

The findings presented here demonstrate the difficulties that organisms are likely to face if relying on phenotypic plasticity when confronting rapid environmental change and instability. Particularly, as the composition and stability of invertebrate prey communities are predicted to alter under climate change (Cao et al., 2020; Hart & Gotelli, 2011), and foraging behaviour of fishes is likely to modify with different temperature conditions (Biro et al., 2007; Sánchez‐Hernández et al., 2021), there is concern over whether phenotypic plasticity really can act as a rapid‐response mechanism that allows adaptation and continued survival in a changing world (Fox et al., 2019). Natural selection can only act upon expressed phenotypes, yet it remains unclear as to what potential selective pressures may be exerted upon widely variable phenotypes of the sort elicited here for the purposes of plastic rescue (sensu Snell‐Rood et al., 2018), nor is it yet known as to what effect environmental instability might have on intraspecific diversity. It is clear, however, that phenotypic responses to diet changes over the time scales tested here proved unpredictable. They suggest that if resource instability were to be similar in future, organisms such as brown trout, may not benefit substantially from plastic rescue.

## AUTHOR CONTRIBUTIONS

**J. Peter Koene:** Conceptualization (equal); formal analysis (lead); investigation (lead); methodology (equal); writing – original draft (lead). **Colin E. Adams:** Conceptualization (equal); methodology (equal); supervision (lead); writing – review and editing (equal).

## CONFLICT OF INTEREST STATEMENT

The authors declare no conflicts of or competing interests.

## Data Availability

All data supporting the results in this paper and its supporting information are freely available at figshare.com: https://doi.org/10.6084/m9.figshare.24080991.
